# The complete chloroplast genome sequence of the medicinal plant *Paris polyphylla* (Melanthiaceae)

**DOI:** 10.1080/23802359.2019.1689194

**Published:** 2019-11-12

**Authors:** Fei-Ya Zhao, Ai-En Tao, Yang Li

**Affiliations:** a School of Medicine, Tourism and Culture College of Yunnan University, Lijiang, China;; b College of Pharmacy and Chemistry, Dali University, Dali, China;; c Editorial Department of Journal, Dali University, Dali, China

**Keywords:** *Paris polyphylla*, chloroplast, Illumina sequencing, phylogeny

## Abstract

*Paris polyphylla* is a medicinal plant commonly used in southwest of China. In this study, we sequenced the complete chloroplast (cp) genome sequence of *P. polyphylla* to investigate its phylogenetic relationship in the genus *Paris*. The chloroplast genome of *P. polyphylla* was 163,533 bp in length with 37.1% overall GC content, including a large single copy (LSC) region of 84,272 bp, a small single copy (SSC) region of 12,899 bp and a pair of inverted repeats (IRs) of 33,181 bp. The cp genome contained 114 genes, including 79 protein coding genes, 30 tRNA genes, and 4 rRNA genes. The phylogenetic analysis indicated *P. polyphylla* was closely related to *P. marmorata*.

*Paris* is a very complex genus of the Melanthiaceae family, which comprises approximately 24 species in the world. Most of them are widespread in Europe and East Asia (Li 1998; Ji et al. 2006). There are 19 species in China (Ji et al. 2006). The roots of plants within this genus have been widely used in traditional Chinese medicine for thousands of years (Jiangsu New Medical College 1977). Among these species, *P. polyphylla* is a unique species widely distributed in southwest China. The roots of this species have been frequently used as the “Paridis Rhizoma” (Namely “Chong-Lou”) for local medicine in these regions, owing to its analgesic, hemostatic, anti-tumor, and anti-inflammatory activities (Jiangsu New Medical College 1977). However, up to now for such medicinal plant, many studies have mainly explored its genetic diversity (ISSR and RAPD) (Zhang et al. 2004; He et al. 2007), the genome information of *P. polyphylla* is too little published in GenBank, so that insufficient comprehensive genomic resource is conducted for it. At present, we report the chloroplast genome sequence of *P. polyphylla* and find its internal relationships within the genus *Paris*, which can provide basic data for further research of genus *Paris* species useful genome resource in China.

Fresh and clean leave materials of *P. polyphylla* were collected from Yunlong county, Yunnan, China (N25°53′40.48″, E99°17′16.92″), and the plant materials and a voucher specimen (No. TAE03) were deposited at Tourism and Culture College of Yunnan University (Lijiang). Total genomic DNA was extracted using the improved CTAB method (Doyle 1987; Yang et al. 2014), and sequenced with Illumina Hiseq 2500 (Novogene, Tianjin, China) platform with pair-end (2 × 300 bp) library. The raw data was filtered using Trimmomatic v.0.32 with default settings (Bolger et al. 2014). Then paired-end reads of clean data were assembled into circular contigs using GetOrganelle.py (Jin et al. 2018) with *Paris rugosa* (No. NC_038170) as reference. Finally, the cpDNA was annotated by the Dual Organellar Genome Annotator (DOGMA; http://dogma.ccbb.utexas.edu/) (Wyman et al. 2004) and tRNAscan-SE (Lowe and Chan 2016) with manual adjustment using Geneious v. 7.1.3 (Kearse et al. 2012).

The circular genome map was generated with OGDRAW v.1.3.1 (Greiner et al. 2019). Then the annotated chloroplast genome was submitted to the GenBank under the accession number MN518849. The total length of the chloroplast genome was 163, 533 bp, with 37.1% overall GC content. With typical quadripartite structure, a pair of inverted repeats (IRs) of 33,181 bp was separated by a small single copy (SSC) region of 12,899 bp and a large single copy (LSC) region of 84,272 bp. The cp genome contained 114 genes, including 79 protein coding genes, 30 tRNA genes, and 4 rRNA genes. Of these, 22 genes were duplicated in the inverted repeat regions, 8 protein-coding genes, and 6 tRNA genes contain one intron, while three genes (*ycf3*, *rps12* and *clpP*) have two introns.

To investigate its taxonomic status, a total of 14 cp genome sequences of the genus *Paris* species were downloaded from the NCBI database used for phylogenetic analysis. After using MAFFT V.7.149 for aligning (Katoh and Standley 2013), jModelTest v.2.1.7 (Darriba et al. 2012) was used to determine the best-fitting model (GTR + G) for the chloroplast genomes. Then Maximum likelihood (ML) analysis was performed by RaxML v.8.2.4 (Stamatakis 2014) with 1000 bootstrap replicates, and two Liliaceae species (*Fritillaria cirrhosa*: KF769143 and *Lilium bakerianum*: NC_035592) were used as outgroups. The results showed that *P. polyphylla* was closely related to *P. marmorata* (Figure 1). Meanwhile, the phylogenetic relationship in the genus *Paris* was consistent with previous studies and this will be useful data for developing markers for further studies.

**Figure 1.**
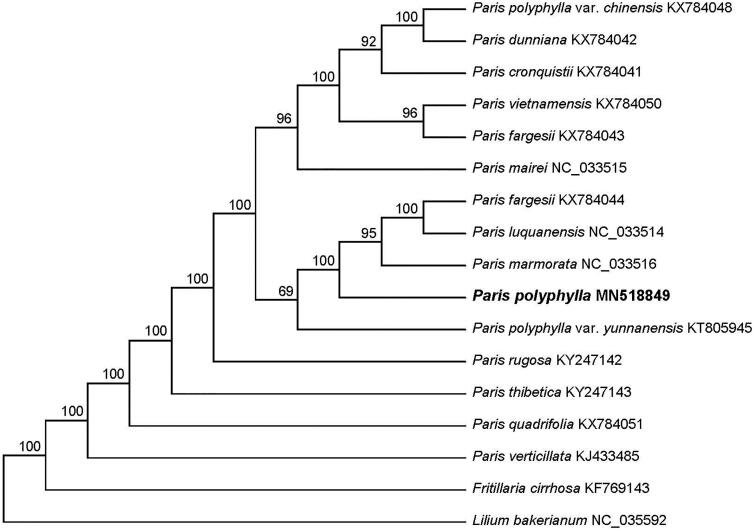
Maximum likelihood (ML) tree of 14 species within the genus *Paris* based on the plastomes using two Liliaceae species as outgroups.
